# Medical student volunteerism and interest in working with underserved and vulnerable populations

**DOI:** 10.1186/s12909-020-02048-x

**Published:** 2020-04-29

**Authors:** Anna Joy G. Rogers

**Affiliations:** College of Medicine, University of Tennessee Health Science Center, 853 Jefferson Avenue, Suite E102, Memphis, TN 38163 USA

**Keywords:** Medical education, Underserved populations, Volunteerism, Ordinal logistic regression

## Abstract

**Background:**

The desire of medical students to eventually work with underserved and vulnerable populations (hereafter ‘service interest’), has been shown to be shaped by individual factors including job satisfaction and financial considerations. School-level factors such as required longitudinal primary care experiences and the availability of extracurricular opportunities to work with underserved patients also affect service interest, but little is known about the impact of student volunteer activities.

**Methods:**

This cross-sectional study gathered data from preclinical medical students via an online questionnaire. The data were linked to academic records, deidentified, and analysed using an ordinal logistic regression model with interest in caring ‘primarily for underserved or vulnerable populations’ as the outcome variable.

**Results:**

Of 121 respondents (33% response rate), 24.8% expressed a definite interest, 55.3% expressed possible interest, and 19.9% expressed no service interest. Intent to work with the underserved was not related to age, sex, race/ethnicity, being from a rural hometown, academic qualifications prior to medical school, or anticipated debt at medical school graduation. Students with no service interest had a higher average academic performance in medical school and plans of subspecialising. When considering volunteerism prior to medical school, students in the highest and middle volunteerism tertiles had 5.68 (95% CI: 1.63, 19.81) and 4.34 (1.32, 14.32) times the odds, respectively, of having definite or possible service interest relative to those who were in the lowest volunteerism tertile, after adjusting for potential confounders. Volunteerism in a student-run clinic for the underserved during medical school was not correlated with service interest.

**Conclusions:**

Medical schools looking to enroll more students interested in working with underserved or vulnerable populations may choose to emphasise applicant premedical volunteerism record in their admissions decisions.

## Background

The convergence of population growth, ageing, and changing healthcare environments has led to a primary care physician shortage [1, 2]. Medically vulnerable and underserved patients are especially disadvantaged as the workforce gap makes recruiting general practitioners to serve these populations even more challenging [3]. Medical schools that train students interested in fulfilling this need are a critical part of the primary care workforce pipeline.

Specialty selection is multifactorial, but related to both individual preferences and school-level factors. Research has shown that job satisfaction, financial considerations, and lifestyle of physicians in the chosen specialty are major contributors to specialty choice [4, 5]. In addition, institutions that have required longitudinal primary care experiences, [6] a school culture that values primary care, [7] and special tracks aimed at recruiting and maintaining students interested in working with underserved populations [8] are more likely to graduate students who choose primary care.

Volunteer service learning activities have been shown to improve student engagement in clinical training [9] and attitudes towards working with underserved populations, [10] while supplementing medical education [11]. However, little is known about how volunteerism prior to medical school admission impacts desire to work with disadvantaged populations, and whether students who have long-term plans of working with underserved patients are more likely to involve themselves in volunteering while in medical school. We hypothesised that greater desire to work with underserved and vulnerable populations would be correlated with higher levels of volunteerism both prior to and during medical school.

## Methods

### Setting and recruitment

Data were gathered at a large School of Medicine^1^ in the Southeastern United States among preclinical medical students. Ethics approval was given by the Institutional Review Board. A questionnaire was designed after conducting an extensive literature review on the factors influencing the decision of medical students to pursue a career in primary care or serve vulnerable populations. The questionnaire was first piloted among 20 third- and fourth-year medical students in their clinical years to assess the clarity and comprehensiveness of the questions, then modified to address deficiencies. All preclinical (first- and second-year) medical students were invited to complete the questionnaire through an in-class announcement and two email reminders. The questionnaire was administered over a one-week period through CheckBox (Checkbox Survey Software, Watertown, Massachusetts, USA), a secure professional questionnaire software tool. Participants gave electronic informed consent and were entered into a drawing for two $50 debit cards.

### Questionnaire

The 31-item questionnaire was designed to assess past volunteering experiences, career interests, and personal demographics. The primary dependent variable, interest in working with underserved or vulnerable populations, was a response of ‘yes,’ ‘maybe,’ or ‘no’ to the question ‘Do you plan to care primarily for an underserved or vulnerable population?’ Those who responded with ‘yes’ are coded as having a ‘definite’ interest in caring for underserved patients; those who responded with ‘maybe’ are coded as having a ‘possible’ interest. The primary independent variable, volunteerism, is a composite score created by combining answers to five related questions assessing whether students had volunteer experience in (a) community service or community health, (b) hospital-based medicine, (c) health disparities, (d) underserved populations, and (e) international development or global health. Participants were asked whether they engaged in these activities at three levels of education – high school, college, and post-college but before medical school. If they did participate, they were asked to rate their involvement as being either low, medium, or high, which contributed one, two, or three points respectively to their composite score. Since there were five avenues for involvement, the composite score at each level of education could range from 0 to 15, and when combining the score at all three educational levels, the overall composite score could range from 0 to 45. Scores were scaled to a maximum of 10 points. Other independent variables included student demographics, personal characteristics, long-term goals, and financial history.

### Student records

Participants gave informed consent for analysis of their deidentified data, including Medical College Admissions Test (MCAT) scores, graduate degree, undergraduate grade point average, and medical school raw grades and percentile ranks as of the end of their preclinical years of medical school. A custodian of student records linked data from the questionnaire to each participant’s academic record and deidentified the data for analysis.

### Statistical analysis

Ordinal logistic regression was used to assess the association between volunteerism and the ordered outcome of interest in working primarily with underserved or vulnerable populations. Ordinal logistic regression for a trichotomous outcome is analogous to logistic regression for a dichotomous outcome. However, it requires a proportional odds assumption, which states that the relationship between each pair of dichotomous outcomes (i.e., definite vs. possible interest and possible vs. no interest) is the same; thus only one odds ratio is sufficient to compare outcome groups.

The data for both years were pooled and analysed using Stata (StataCorp, 2017, Stata Statistical Software: Release 15. College Station, TX: StataCorp LP). Simple t-tests and one-way ANOVA calculations were used to construct Table 1 proportions and calculate statistical significance. The proportionality assumption was tested using Stata commands *omodel* and *brandt,* with nonsignificant test statistics demonstrating that the proportional odds assumption was not violated. A proportional odds ratio with a trichotomous ordered outcome variable was calculated for all ordinal logistic regression models. A *p*-value of less than 0.05 was considered strong evidence against the null hypothesis.

**Table 1:** Medical student characteristics by desire to care primarily for an underserved or vulnerable population

	Answers to the question, ‘Do you plan to care primarily for an underserved or vulnerable population?’			
	No *n* = 24 (%)	Maybe *n* = 67 (%)	Yes *n* = 30 (%)	*P* value
*Characteristic*				
Age	23.7	23.3	24.1	0.197
Sex (Female)	9 (38)	31 (46)	17 (57)	0.366
Race/Ethnicity (Nonwhite)	3 (14)	10 (16)	8 (32)	0.191
Hometown (Rural)	12 (54)	35 (53)	17 (56)	0.272
Active in religion/philosophy	12 (55)	35 (54)	17 (57)	0.976
At least one parent physician	8 (35)	12 (18)	5 (17)	0.214
*Academic qualifications*				
Master’s degree	1 (8)	6 (9)	6 (20)	0.160
MCAT Average Score	30.8	30.6	29.2	0.118
Undergraduate GPA Average	3.78	3.77	3.76	0.914
Medical School Percentile Average	62.6	50.9	44.7	0.065
*Future plans*				
Long-term goals				0.038*
Primary care	3 (16)	10 (18)	12 (40)	
Other specialty/ Subspecialise	11 (58)	25 (45)	6 (20)	
Undecided	5 (26)	21 (38)	12 (40)	
Plan to locate in underserved area	2 (8)	20 (30)	19 (63)	< 0.001*
*Finances*				
Undergraduate debt	3,049	5,080	12,910	0.069
Anticipated medical school debt	109,102	114,395	86,000	0.300
Anticipated total debt	112,283	117,967	99,339	0.875
Belief that debt at graduation influence career choice	8 (35)	25 (38)	6 (20)	0.218
*Volunteerism (composite scores out of 10)* During medical school	4.0	5.0	3.6	0.588
Before medical school (premedical)	2.1	3.2	4.3	< 0.001*
High school	1.3	2.7	3.2	0.004*
College	3.0	4.6	5.3	0.008*
After College	1.9	2.0	3.8	0.010*

*MCAT* Medical College Admissions Test

*Indicates *p* value less than 0.05

## Results

A total of 121 medical students completed the questionnaire. The response rates of the Classes of 2016 and 2017 were 42 and 27%, respectively, with a pooled response rate of 33%. Of the students in the sample, the average age was 23.5 years (range: 21–34), 47% were female, and 20% were non-Whites. Of all respondents, 24.8% expressed a definite interest, 55.3% expressed possible interest, and 19.9% expressed no interest in working with underserved and vulnerable populations (hereafter ‘service interest’).

Table 1 shows the baseline distribution of medical student characteristics by service interest. Students with definite or possible service interest were similar to those with no interest in terms of age, sex, race/ethnicity, and whether or not they were from rural hometowns. They were also similar in terms of academic qualifications prior to medical school, as seen by similar MCAT scores and undergraduate grade point averages. However, by the end of their preclinical years in medical school, students with no service interest had a higher academic standing, as shown by average percentile scores that were 17.9 and 11.7 points higher respectively, when compared to students with definite or possible service interest, although the result was not statistically significant. Similarly, they were significantly more likely to desire to specialise outside of primary care, and far less likely to want to locate their practice in an underserved area. There was no difference between the three groups in terms of their financial situations, such as whether they believed that debt at graduation would influence their career choices.

The primary independent variable of volunteerism before medical school differed significantly across the three service interest levels. Those with definite service interest had an average composite score 1.1 points higher (out of ten) than students with possible interest, who in turn had an average composite score 1.1 points higher than those with no interest. This trend remained even after stratification into educational levels of high school, college, and after college. There was no difference between the groups in terms of volunteerism at a student-run free clinic during medical school.

To understand factors associated with service interest, we assessed five independent variables that in Table 1 had a *p* value less than the threshold of 0.10 or that have been shown in the literature to impact likelihood of such interest. These variables – premedical volunteerism, medical school percentile, hometown, long-term goals, and desire to locate practice in an underserved area – met the proportional odds assumption. Odds ratios greater than 1 (the reference value, ‘ref’) indicate that the level of the independent variable is associated with higher service interest, proportionally for those with possible and definite interest. As shown in Table 2, students in the highest premedical volunteerism tertile had 5.92 (95% Confidence Interval [CI]: 2.31, 15.13) times the odds of having possible service interest (vs. no interest) relative to those who were in the lowest volunteerism tertile. Due to the proportional odds assumption having been met, students in the highest volunteerism tertile also had 5.92 times the odds of definite service interest (vs. possible interest) relative to those in the lowest volunteerism tertile. The unadjusted proportional odds ratio (POR) was statistically significant at the alpha = 0.05 level for medical school percentile, long-term goals, and desire to locate practice in an underserved area; suggesting that service interest was correlated with lower performance during medical school and long-term goals that were directed towards primary care.

**Table 2:** Unadjusted and adjusted proportional odds ratios of medical students wanting to ultimately care for an underserved or vulnerable population

Medical student characteristics	Proportional odds ratios (ORs) of medical students wanting to ultimately care for an underserved or vulnerable population		
	Crude OR (95% CI)	Adjusted^a^ OR (95% CI)	Adjusted^b^ OR (95% CI)
Premedical volunteerism composite variable^			
Lowest tertile	(ref)	(ref)	(ref)
Middle tertile	3.94 (1.59, 9.78)	2.72 (0.99, 7.36)	4.34 (1.32, 14.32)
Highest tertile	5.92 (2.31, 15.13)	4.33 (1.51, 12.40)	5.68 (1.63, 19.81)
Being from a rural hometown*	1.91 (0.91, 4.00)		1.27 (0.44, 3.67)
Medical school academic percentile^			
Lowest tertile	(ref)		(ref)
Middle tertile	1.29 (0.54, 3.06)		2.40 (0.80, 7.19)
Highest tertile	0.33 (0.14, 0.79)		0.58 (0.18, 1.83)
Long-term career goals^			
Other specialty/	(ref)		(ref)
Subspecialise			
Undecided	2.45 (1.02, 5.82)		1.64 (0.59, 4.54)
Primary care	4.39 (1.60, 12.05)		1.95 (0.57, 6.73)
Desire to locate practice in underserved area^			
No	(ref)		
Maybe	12.49 (4.79, 43.75)		
Yes	47.40 (13.67, 164.3)		

*CI* Confidence interval; *ref.* Reference; *MCAT* Medical College Admissions Test.

^Crude proportional odds ratio had a *p*-value less than 0.01.

*Crude proportional odds ratio had a *p*-value of 0.086

^a^Model adjusted for age, MCAT score, race/ethnicity, having a graduate degree, and undergraduate debt

^b^ In addition to adjusting for the variables mentioned above, this model adjusted for long-term goals, hometown, and medical school percentile. Due to a high level of correlation between service interest and wanting to locate practice in an underserved area, the latter variable was not included in final adjusted models

Table 2 also shows the adjusted models for the association between premedical volunteerism and service interest. After adjusting for the potential confounders of age, MCAT score, race/ethnicity, and having a graduate degree, the POR was attenuated to 4.33 (95% CI: 1.51, 12.40) when comparing the highest to the lowest tertiles of premedical volunteerism, and 2.72 (95% CI: 0.99, 7.36) when comparing the middle to lowest tertiles. After adjusting for the additional potential confounding of long-term goals, hometown, and percentile, the relationship between premedical volunteerism and service interest remained statistically significant.

## Discussion

The current shortage of physicians serving vulnerable and underserved populations is projected to worsen with population growth and ageing. Without an understanding of the factors driving career decision-making of medical school graduates, simply educating and training more physicians will not meet the growing demand. Medical school admissions committees play an important role in the production of primary care physicians, [12, 13] but may not be aware of characteristics predictive of long-term interest in serving vulnerable and underserved patient populations.

Utilizing data from a questionnaire and student academic records, we found that preclinical medical students with a definite interest in caring for the underserved had volunteered significantly more often and in a wider variety of settings *prior to* medical school than students with possible service interest, who subsequently had more volunteerism than students with no service interest. The relationship between premedical volunteerism and service interest remained significant after stratifying volunteerism at all educational levels – high school, college, and after college. After adjusting for potential confounders such as student demographics, long-term goals, and medical school academic grades, the relationship between premedical volunteerism and interest in serving underserved and vulnerable populations was attenuated but remained statistically significant.

Unexpectedly, service interest was not predictive of volunteerism at a student-run clinic for underserved populations *during* medical school, as has been reported elsewhere [6, 10]. This may be because the clinic allows for preclinical students to get hands-on experience with patients before they are officially in their clerkship years. Thus, many students may take advantage of this opportunity, regardless of their interest in primary care or working with vulnerable patients.

Secondary findings from this study suggest that students with a service interest are not significantly different in terms of age, sex, race/ethnicity, hometown, and academic performance prior to medical school. Additionally, there did not seem to be an effect of projected total debt on desire to work with underresourced populations, corroborating other research that financial compensation for debt repayment does not play a large role in the decision-making [14]. However, the data show that medical school grades are higher among students without a service interest. This same group of students is more likely to desire to a non-primary care specialty or to subspecialise. Thus, the pressure to perform well academically in order to get into their chosen medical specialty may provide incentive for those without a service interest to distinguish themselves though their grades.

While this is the first known study to demonstrate an association between volunteerism prior to medical school and interest in working with vulnerable populations, other studies have supported the importance of such volunteerism to the professional development of medical students. One study suggested that health professional students who engage in service-learning opportunities during medical school may not only develop patient interaction and clinical skills, but also an understanding of health disparities [15]. Another study found that students who were heavily engaged in volunteering during medical school were on par academically with those who were unengaged or less engaged, suggesting that volunteerism may complement rather than compete with the medical school curriculum [16].

The strengths of this study include comprehensive assessment of the main independent variable of volunteerism; availability of data on a wide range of demographic, experiential, and academic factors; and the anonymous nature of the questionnaire, which reduced the likelihood of social desirability bias impacting student answers. This study had several limitations. First, there may have been self-selection of students who chose to answer this questionnaire, given that the pooled response rate was 33%, thus making it difficult to generalise the results to the student body. Second, the cross-sectional nature of the study makes it difficult to understand the temporal nature of the relationships identified. Third, while it is likely that students who profess an interest in working with the underserved will eventually do so, [17] this study did not follow students to determine their actual career choices. Fourth, both recall bias and social desirability bias, which may have been influenced by the extrinsic motivation of a drawing for gift cards, are potential considerations. However, we limited the likely impact of these by blinding respondents to the hypothesis and using a small-value incentive for participation. Finally, we lacked comparative information on students who did not complete the survey (ie. overall target population), as well as on medical student volunteer efforts outside of the student-run clinic for underserved patients.

## Conclusion

Medical school admissions committees seeking to address the growing shortage of physicians who choose to serve vulnerable and underserved patient populations should consider the volunteerism history of applicants. Our study suggests that level of volunteerism may correlate positively with level of service interest. Future research should elucidate the ways in which volunteerism prior to medical school influences desire to work with vulnerable populations and should determine whether expressed service interest translates into actual career decisions of working in underserved areas. A greater understanding of the factors influencing medical student commitment to working in areas of health care professional shortage will help meet the growing demand for physicians.

## Data Availability

The datasets generated and/or analysed during the current study are not publicly available due to small class sizes and sensitivity of data points but are available from the corresponding author on IRB approval of request.

## Abbreviations

MCAT: Medical college admissions test
POR: Proportional odds ratio

## Footnotes

1 Institution is not specified to protect the privacy of student and institutional data, but is available through correspondence with the author.
